# Differential effects of RYGB surgery and best medical treatment for obesity-diabetes on intestinal and islet adaptations in obese-diabetic ZDSD rats

**DOI:** 10.1371/journal.pone.0274788

**Published:** 2022-09-22

**Authors:** Ananyaa Sridhar, Dawood Khan, Mahmoud Abdelaal, Jessie A. Elliott, Violetta Naughton, Peter R. Flatt, Carel W. Le Roux, Neil G. Docherty, Charlotte R. Moffett

**Affiliations:** 1 Biomedical Sciences Research Institute, School of Biomedical Sciences, Ulster University, Coleraine, Northern Ireland, United Kingdom; 2 Diabetes Complications Research Centre, School of Medicine, Conway Institute of Biomolecular and Biomedical Research, University College Dublin, Dublin, Ireland; 3 Department of Surgery, Trinity Centre for Health Sciences and St. James’s Hospital, Dublin, Ireland; Institute of Metabolic Science, University of Cambridge, UNITED KINGDOM

## Abstract

Modification of gut-islet secretions after Roux-En-Y gastric bypass (RYBG) surgery contributes to its metabolic and anti-diabetic benefits. However, there is limited knowledge on tissue-specific hormone distribution post-RYGB surgery and how this compares with best medical treatment (BMT). In the present study, pancreatic and ileal tissues were excised from male Zucker-Diabetic Sprague Dawley (ZDSD) rats 8-weeks after RYGB, BMT (daily oral dosing with metformin 300mg/kg, fenofibrate 100mg/kg, ramipril 1mg/kg, rosuvastatin 10mg/kg and subcutaneous liraglutide 0.2mg/kg) or sham operation (laparotomy). Insulin, glucagon, somatostatin, PYY, GLP-1 and GIP expression patterns were assessed using immunocytochemistry and analyzed using ImageJ. After RYGB and BMT, body weight and plasma glucose were decreased. Intestinal morphometry was unaltered by RYGB, but crypt depth was decreased by BMT. Intestinal PYY cells were increased by both interventions. GLP-1- and GIP-cell counts were unchanged by RYGB but BMT increased ileal GLP-1-cells and decreased those expressing GIP. The intestinal contents of PYY and GLP-1 were significantly enhanced by RYGB, whereas BMT decreased ileal GLP-1. No changes of islet and beta-cell area or proliferation were observed, but the extent of beta-cell apoptosis and islet integrity calculated using circularity index were improved by both treatments. Significantly decreased islet alpha-cell areas were observed in both groups, while beta- and PYY-cell areas were unchanged. RYGB also induced a decrease in islet delta-cell area. PYY and GLP-1 colocalization with glucagon in islets was significantly decreased in both groups, while co-staining of PYY with glucagon was decreased and that with somatostatin increased. These data characterize significant cellular islet and intestinal adaptations following RYGB and BMT associated with amelioration of obesity-diabetes in ZDSD rats. The differential responses observed and particularly those within islets, may provide important clues to the unique ability of RYGB to cause diabetes remission.

## Introduction

Obesity and its associated metabolic aberrations have been a prevalent topic of research for the past few decades. Bariatric surgery has proven to be the most successful treatment option, recording significantly greater weight loss compared to more conservative treatments [1]. The most effective and common type of bariatric surgery is Roux-en-Y gastric bypass (RYGB), that also leads to remissions in Type 2 Diabetes Mellitus (T2DM) [2]. Although the precise mechanism behind this outcome is unclear, extensive research suggests major improvements of insulin sensitivity and islet cell function with improved glucose-stimulated insulin secretion. Additionally, improved utilization of glucose and enhanced release of gut hormones play key roles in helping to achieve sustained benefits from surgery [3–6].

Many studies have been performed on changes of circulating gut hormones concentrations following RYGB [7–11]. Despite some contradictory findings, hormones secreted predominantly by enteroendocrine cells (EECs) of the lower small intestine, that now feeds directly from the stomach, are increased including GLP-1, oxyntomodulin, CCK and secretin [3, 12–16]. In contrast, ghrelin from the restricted stomach and GIP released from the bypassed K-cells of the duodenum may be decreased [17–19]. Previous studies also reported elevated levels of post-prandial PYY in rat and human models of RYGB [20, 21]. Interestingly, one study observed no difference in weight loss between gastric bypass and sham operated PYY knockout mice [22]. The alimentary and biliopancreatic limb in humans exhibited an increase in the number of PYY and GLP-1 positive cells after RYGB surgery [23]. The increase of circulating GLP-1 which is observed consistently is believed to be a key player in the metabolic benefits mediated through inhibition of gastric motility, induction of satiety, suppression of glucagon and stimulation of insulin release [24–26]. Nevertheless, increased release of PYY from the same intestinal L-cells as GLP-1 has recently been proposed to play an important role. Like GLP-1, PYY(1–36) is subject to N-terminal degradation by DPPIV, producing PYY(3–36) which induces satiety via central action [27]. The intact peptide PYY(1–36) lacks effects on feeding but serves to induce beta-cell mass and rest of these insulin secreting cells [28]. Although less well known, substantial amounts of PYY are also present in pancreatic islets where it may play a largely unrecognized local regulatory paracrine role [29].

Interestingly, changes in insulin, glucagon, and possibly somatostatin, produced by the islet endocrine cells appear to contribute to remission of T2DM [3]. Fasting levels of insulin decrease by up to 60% post-RYGB due to improvements in insulin resistance, and clearance, which are promoted by substantial weight loss [19]. Post-RYGB, beta-cell response to glucose is substantially enhanced [30]. However, possible changes in beta-cell mass after surgery remain debatable as some studies suggest an increase, whilst others report either a decrease or no change [20, 31–35]. Similarly, studies on islet alpha and delta cell populations after bypass surgery have also reported inconclusive results [32], in line with inconsistency in measured levels of circulating glucagon and somatostatin [19, 36–40]. Previous studies in our laboratory suggest adaptation of PYY and GLP-1 within the islets under conditions of cellular stress [29, 41]. In GLP-1 receptor knockout mice, there was comparable weight loss and improvement of glucose tolerance compared with control animals after RYGB [42]. In addition, RYGB significantly reduced plasma GLP-1 levels with GLP-1 receptor blockade resulting in a 2-3-fold reduction of insulin secretion [37]. Correspondingly, GIP has presented inconsistent measurements post-RYGB with studies reporting increase, decrease and no change [36, 43–45]. Reduced mRNA levels of GIP were observed in the intestine post RYGB in human subjects. Interestingly, same study observed increase in GIP positive cell density in RYGB group [23]. Taken together, it is evident that gut-hormonal adaptation is vital under conditions of cellular stress and further studies are required for a definitive conclusion of their role in the beneficial impact of gastric bypass surgeries.

In the present study, we examined the relative effects of RYGB, and a body weight and metabolic control matched medical intervention on distribution of key gut hormones within the intestine and pancreatic islet size, stress and hormone profiles in obese-diabetic ZDSD rats. Comparison of these effects may help to reveal what RYGB does over and above best medical treatment using metformin/liraglutide to ameliorate diabetes.

## Methods

### Animals

Animal studies, including surgeries were carried out with male Sprague Dawley (SD) and Zucker Diabetic Sprague Dawley (ZDSD) rats obtained at 14 weeks of age from CrownBio (Belgium). Experiments were conducted under governmental project license (Health Products Regulatory Authority–AE18982/Po84). Experiments were carried out under the UK Animals (Scientific Procedures) Act 1986 as well as being approved by the University College Dublin Animal Research Ethics Committee and Ulster University Animal Welfare and Ethical Review Body (AWERB). Rats were housed 2/cage for the ZDSD and 3-4/cage for the SD at 22±2°C, 35% humidity and 12h light/dark cycle. All rats were fed a standard chow Purina 5008 diet (4.36 kcal/g, carbohydrate 57.7%, protein 23.6%, fat 6.7% from Nestle Purina, St. Louis, MO) with ad-libitum access to drinking water. 16 animals were divided equally into four groups for the study: control (SD), sham (ZDSD), RYGB (ZDSD), and best medical treatment (BMT—ZDSD) involving daily co-administration of oral metformin (300mg/kg), fenofibrate (100mg/kg), ramipril (1mg/kg), rosuvastatin (10mg/kg) and subcutaneous liraglutide (0.5mg/kg). The basic study design was similar to that described previously [46]. Body weight and blood glucose were monitored weekly before and at termination of treatments. Animals were euthanized in the non-fasted state at the same time of day (10:00–14:00h) to minimize possible variation. Tissues (pancreas and intestine) were harvested 8 weeks after treatment.

### RYGB and sham surgery

The rats in the sham and BMT groups underwent sham surgery alongside RYGB surgery that were performed at 29 weeks of age. Animals were treated for 7 days prior to surgery with long-acting insulin degludec (Tresiba®, Novo Nordisk A/S), with the aim of achieving blood glucose 6–9 mmol/l. The animals were anesthetized using isoflurane. A single dose of enrofloxacin (7.5mg/kg—Baytril, Bayer) was administered at induction of anesthesia and buprenorphine (0.02mg/kg–Animalcare Limited) administered at the end of surgery for postoperative pain. Lacrilube eye ointment was applied to prevent corneal dryness during anesthesia. Following supine positioning, a 2cm upper midline laparotomy incision was made. The proximal jejunum was divided 10 cm distal to the duodenojejunal flexure to create the biliopancreatic and alimentary limbs. A side-to-side jejunojejunal anastomosis was then formed using a single layer technique with interrupted 6–0 prolene sutures approximately 30cm from the ileocaecal valve. The lesser curve vessels were carefully dissected and the gastric cardia divided to create a small gastric pouch (max 3 ml volume). This was achieved by transecting the stomach approximately 5mm below the gastro-oesophageal junction. The gastric remnant was closed with interrupted 6–0 prolene. An end-to-side gastrojejunal anastomosis was then formed using 6–0 prolene sutures. 5 ml of warmed, sterile saline (0.9%, 3.5 ml each side) was administered intra-peritoneally before closure. The linea alba was closed with continuous 4–0 Vicryl (Ethicon, Inc.), and the skin closed with subcuticular 4–0 Vicryl. The rats were placed into a clean cage with a warming pad and towel to recover from anesthesia. When fully recovered, they were returned to a clean home cage. For the sham operation, an upper midline laparotomy incision was made, the small bowel and stomach exposed, and the abdomen was then closed, as described above.

#### Food restriction and medical treatment in BMT group

Following surgery, food for the BMT group was reduced to achieve 10% weight loss in 3 weeks. All rats received 16g of standard chow (Purina 5008). The amount of food given was adjusted according to their weight change. In addition, they received metformin hydrochloride (300mg/kg). Blood glucose was measured after 10 days following which the rats received oral rosuvastatin (10mg/kg), fenofibrate (100mg/kg) and ramipril (1mg/kg). Subcutaneous Liraglutide treatment began when 10% weight loss was achieved and the dose was titrated from 0.025 to 0.5mg/kg over 14 days. Medications were introduced two weeks after surgery once the rats were established on a regular chow diet. Metformin monotherapy was introduced for the first two days to monitor for adverse responses, including anorexia. The remaining medications (fenofibrate, ramipril, rosuvastatin and liraglutide) were commenced thereafter when no adverse response was observed.

#### Post-surgical care

Post-surgery, the animals were housed for 3 days without bedding, to prevent ingestion of bedding material and anastomotic obstruction. Only water was available ad libitum at the day of surgery. There was approximately 30% mortality in the RYGB group while no deaths were encountered in the other groups of rats. For a period of five consecutive days post-operation, the animals were treated with enrofloxacin (7.5mg/kg) and one to three days buprenorphine (0.05mg/kg), as required. On day one and two post-surgery, the rats received 20ml and 30 ml of liquid diet respectively (Ensure® Plus, Vanilla, 1.5kcal/ml, 16.7% protein, 29.5% fat, 53.8% carbohydrate). On day three they were returned onto normal bedding. On days 6–7 post surgery, all rats received standard chow (Purina 5008). During the first ten days post-surgery, all rats were monitored daily for weight loss, food and water intake, pain and sings of infection. If the weight loss was > 30% the animal was euthanized.

### Tissue processing

Pancreatic and intestinal tissues from RYGB, BMT, sham and SD rats were fixed for ~48 hours in paraformaldehyde solution (4% w/v in phosphate buffered saline) to preserve cellular architecture by cross-linking proteins. The tissues were then processed in an automated tissue processor which involved dehydrating tissues in 70% to 100% ethanol, followed by xylene immersion to remove wax before paraffin embedding. The tissues were then sliced into 5μm slices and placed on poly-l-lysine coated slides [47].

### Immunohistochemistry

To assess immunoreactive staining for insulin, glucagon, PYY, somatostatin, GLP-1, Ki-67 and TUNEL, sections were dewaxed in histoclear for 30 mins before being rehydrated with decreasing concentrations of ethanol. The sections were blocked with 2.5% bovine serum albumin (BSA) and then incubated with a primary antibody (Table 1) for the respective peptide overnight. On day 2, the sections were rinsed in phosphate-buffered saline (PBS) twice and incubated with secondary antibody (Alexa Fluor^®^ 594 for red and Alexa Fluor^®^ 488 for green; Table 1) for 1 hour at 37°C. After two more PBS washes, they were incubated with DAPI for 15 mins at 37°C followed by a last set of washes with PBS [47]. Finally, the sections were mounted using antifade and coverslips. Stained sections were viewed at 40x magnification using an Olympus IX51 inverted microscope and photographed using a DP70 digital camera system.

**Table 1:** Target, host and source of primary and secondary antibodies employed for immunofluorescent islet histology studies.

**Primary antibodies**			
**Target**	**Host**	**Dilution**	**Source**
Insulin	Mouse	1:500	Abcam, ab6995
Glucagon	Guinea pig	1:4	Raised in-house PCA2/4
PYY	Rabbit	1:500	Abcam, ab22663
GLP-1	Rabbit	1:4	Raised in-house XJIC8
SST	Rat	1:500	Biorad, 8330–009
GIP	Rabbit	1:4	RIC34/111J, kindly donated by Professor L Morgan, Guildford, UK
Ki-67	Rabbit	1:200	Abcam, ab15580
**Secondary antibodies**			
**Host and target**	**Reactivity**	**Dilution**	**Fluorescent dilution and source**
Goat IgG	Mouse	1:500	Alexa Flour 594, Invitrogen, UK
Goat IgG	Guinea pig	1:500	Alexa Flour 488, Invitrogen, UK
Goat IgG	Rabbit	1:500	Alexa Flour 594, Invitrogen, UK
Goat IgG	Rat	1:500	Alexa Flour 488, Abcam

### Image analysis

The Cell^F software was used to analyse images to assess islet area, beta- and alpha-cell area, percentage of beta- and alpha- cells as well as percentage of peptide positive cells. To calculate islet distribution, islets were classified into small (<10,000 μm^2^), medium (10,000 to 25,000 μm^2^) and large (>25,000 μm^2^). To assess colocalization, insulin, glucagon, PYY, somatostatin and GLP-1 positive cells were calculated and their distribution with respect to the other stained peptide was quantified and compared. For beta-cell proliferation, Ki-67 positive cells were counted whereas for apoptosis, TUNEL positive cells were counted. Image J software was used to measure islet, beta- and alpha-cell area and count positive cells for graphical representation.

### Biochemical analyses

Non-fasting plasma glucose was measured using Contour® Glucose strips (Bayer Ltd, Dublin Ireland). Blood was obtained by puncturing the lateral tail vein and measured using the Bayer Contour Blood Glucose Meter. The maximum detection limit for glucose was 33.3 mmol/L. Values above that could not be measured and were thus included in statistical analyses as 33.3 mmol/L. Intestinal tissues were excised and snap frozen immediately in liquid nitrogen and stored at -80°c. Tissues were homogenized using RIPA buffer and 0.1% protease inhibitor. Homogenized tissues were centrifuged at 664g for 20mins at 4°C. Supernatant was used to perform Bradford assay to determine protein concentration. Remaining supernatant was stored at -80°C for further analysis. Biochemical analyses were carried out for total PYY (rat PYY ELISA, ORB441862-BOR, Stratech Scientific), GLP-1 (GLP-1 total ELISA, EZGLP-1T-36K, Millipore) and GIP (rat/mouse GIP ELISA, EZRMGIP-55K, Millipore) by specific enzyme linked immunoassays following the manufacturers’ instructions. All commercial assay kits have been shown to exhibit a high degree of specificity.

### Statistical analysis

GraphPad PRISM (version 5.0) software was used to perform statistical analysis. There was no inclusion and exclusion criteria applied. A one-way ANOVA with Bonferroni post hoc test was used for comparative analysis between groups. Values are expressed as mean ± S.E.M. Groups of data were considered to be significant if p<0.05.

## Results

### Plasma glucose and body weight after RYGB or BMT

**Fig 1:**
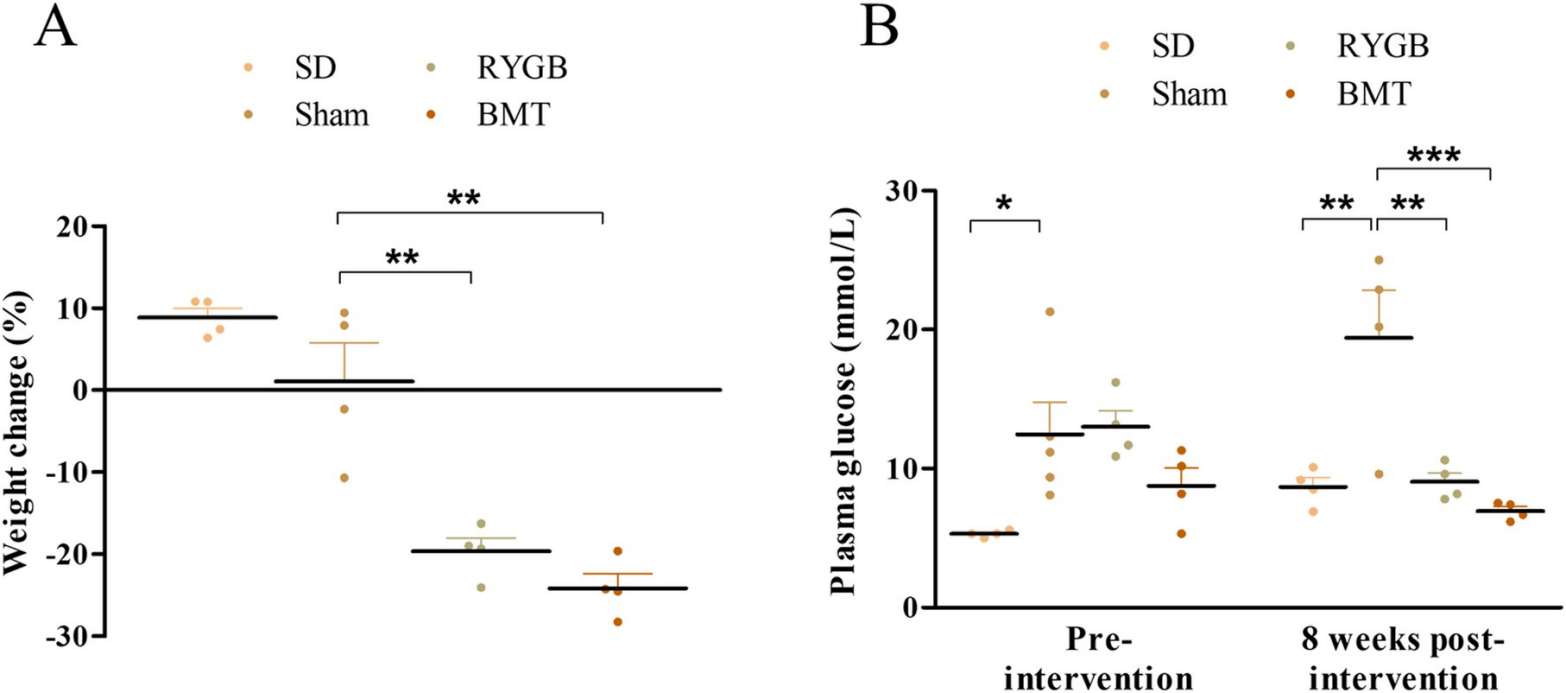
Effects of RYGB, BMT and sham surgery on body weight and non-fasting plasma glucose of ZDSD rats. (A) % Weight change at 8-weeks post-intervention (B) Plasma glucose at 8-weeks post-intervention. Normal SD rats are included for comparison. Values are mean ± SEM (n = 4) with statistical significance evaluated using one-way ANOVA. *p<0.05, **p<0.01, ***p<0.001 compared to sham operated rats.

As previously reported from renal end-point focused studies [46], RYGB significantly reduced body weight and plasma glucose in male ZDSD rats (Fig 1A and 1B). Percentage weight change after 8 weeks of surgery was significantly (p<0.01) lower in the RYGB and BMT groups compared to sham (Fig 1A). Plasma glucose was improved significantly (p<0.01 to p<0.001) post-intervention in RYGB and BMT groups compared to sham (Fig 1B). Prior to surgery, plasma glucose in sham group was significantly (p<0.05) higher to the SD group. This indicates that SD rats are imperfect as true controls, being not genetically identical to their ZDSD counterparts. However, having the SD group helps us understand if RYGB or BMT can restore hormonal profiles close to that of non-obese, non-diabetic animals. The most pertinent and valid comparisons are therefore made between the two experimental groups versus sham controls.

### Ileum morphology after RYGB or BMT

**Fig 2:**
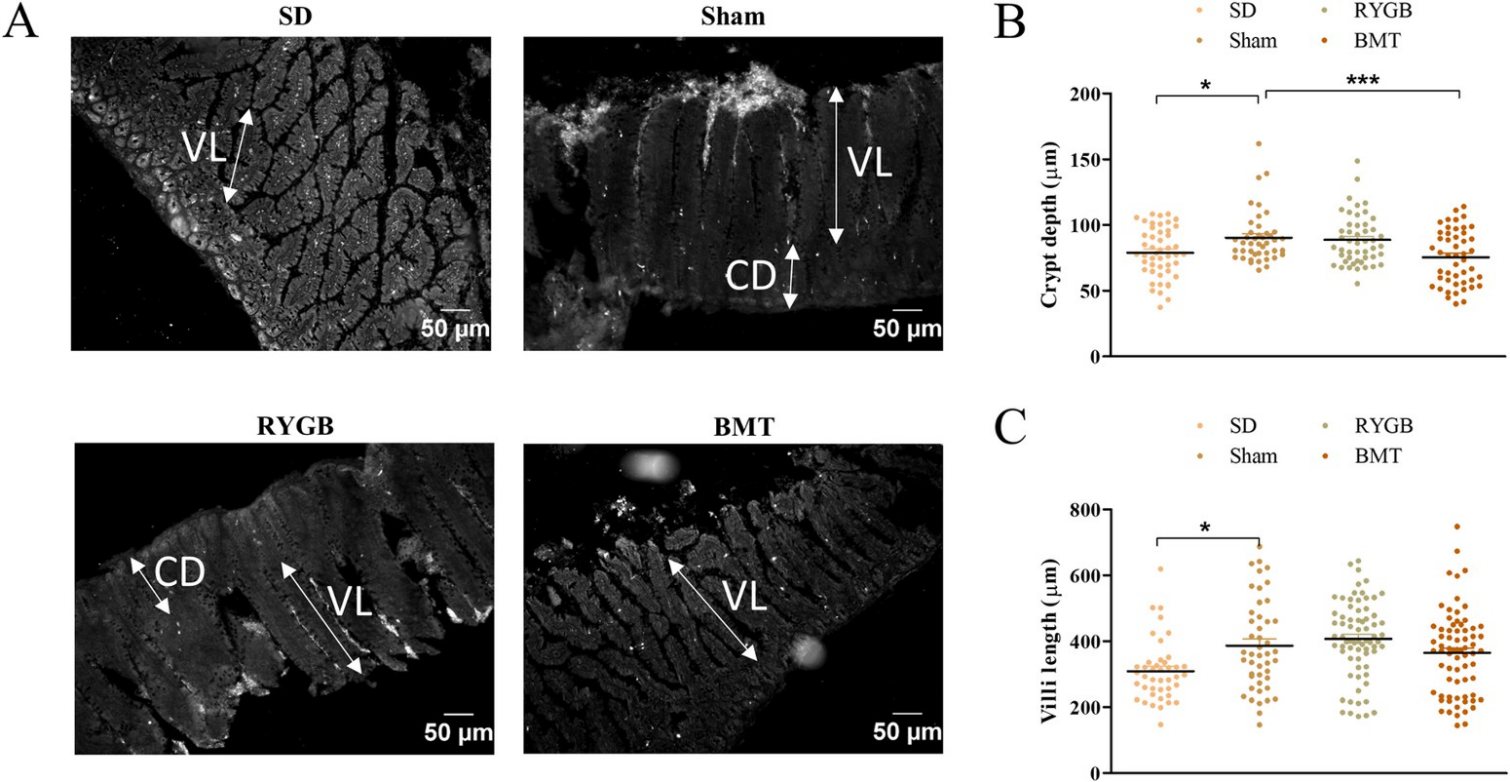
Effects of RYGB, BMT and sham surgery on intestinal morphology in ileum of ZDSD rats 8 weeks post-intervention. (A) Representative images showing cross-section of the ileum; arrows indicate villi length (VL) and crypt depth (CD). Quantification of morphological changes in (B) crypt depth and (C) villi length are shown. Normal SD rats are included for comparison. Values are mean ± SEM (n = 4) with statistical significance evaluated using one-way ANOVA. *p<0.05, ***p<0.001 compared to sham rats.

Representative images of ileum from RYGB, BMT, sham and SD rats are shown (Fig 2A). RYGB did not cause any significant changes in the gross morphology of the ileum with respect to crypt depth and villi length (Fig 2B and 2C). However, BMT caused a significant (p<0.001) decrease in crypt depth compared to sham. Also, villi length and crypt depth were significantly (p<0.05) shorter in SD controls relative to sham.

### GIP-cells in the ileum after RYGB or BMT

**Fig 3:**
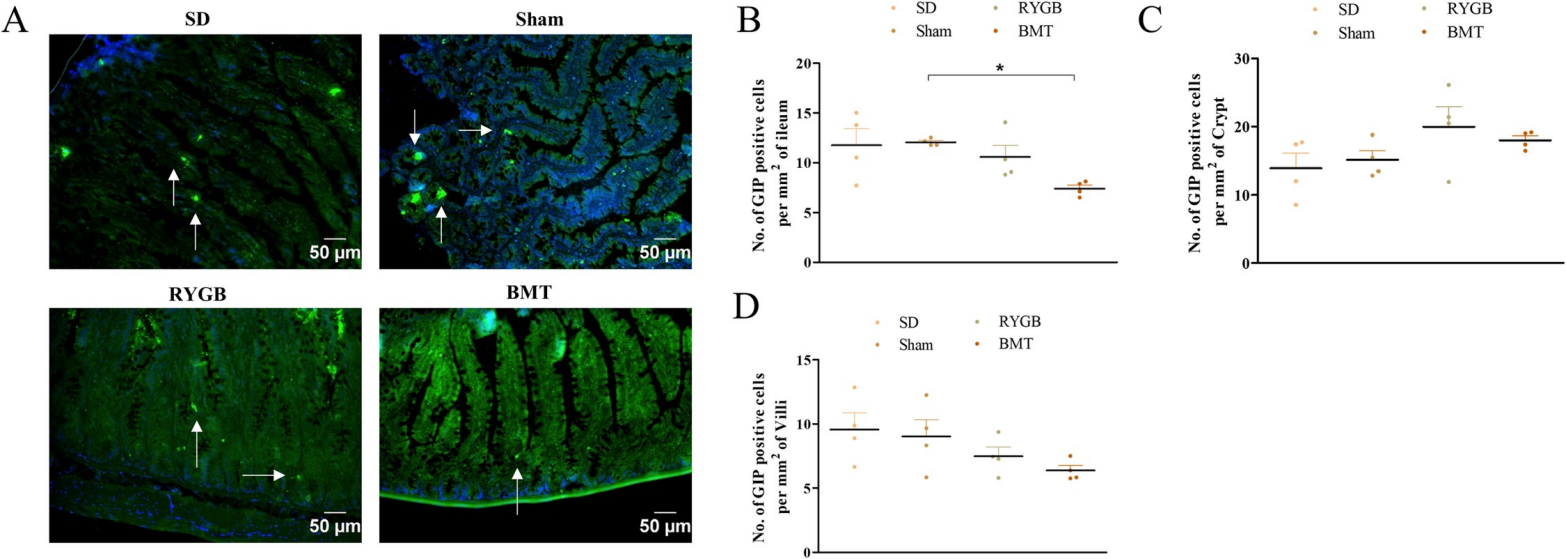
Effects of RYGB, BMT and sham surgery on GIP positive cell distribution in the ileum of ZDSD rats. (A) Representative images showing GIP (green); arrows indicate GIP positive cells. Nuclei are demonstrated using DAPI staining (blue). (B) number of GIP positive cells per mm^2^ of ileum, (C) number of GIP positive cells per mm^2^ of crypt and (D) number of GIP positive cells per mm^2^ of villi analyzed in ileum sections. Normal SD rats are included for comparison. Values are mean ± SEM (n = 4) with statistical significance evaluated using one-way ANOVA. *p<0.05 compared to sham rats. Analyses carried out on ~200 cells per group.

Representative images of ileum from RYGB, BMT, sham and SD rats stained for GIP are shown (Fig 3A). RYGB did not significantly affect the number of GIP positive cells per mm^2^ of ileum, crypt and villi (Fig 3B–3D) compared to sham. A similar situation was observed in the SD group relative to sham. Interestingly, the BMT group had a significantly (p<0.05) decreased number of GIP positive cells per mm^2^ of ileum but not in the crypt and villi individually, when compared to sham.

### GLP-1 cells in the ileum after RYGB or BMT

**Fig 4:**
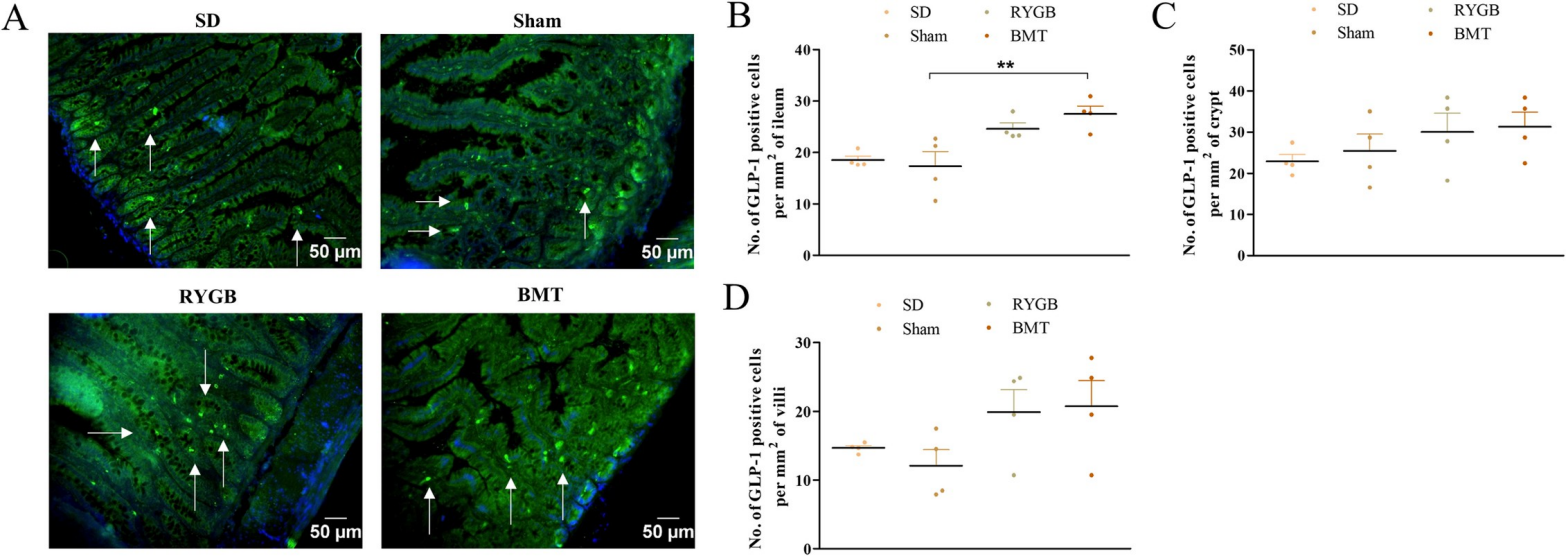
Effects of RYGB, BMT and sham surgery on GLP-1 positive cell distribution in the ileum of ZDSD rat intestine. (A) Representative images showing GLP-1 (green); arrows indicate GLP-1 positive cells. Nuclei are demonstrated using DAPI staining (blue). (B) number of GLP-1 positive cells per mm^2^ of ileum, (C) number of GLP-1 positive cells per mm^2^ of crypt and (D) number of GLP-1 positive cells per mm^2^ of villi analyzed in ileum sections. Normal SD rats are included for comparison. Values are mean ± SEM (n = 4) with statistical significance evaluated using one-way ANOVA. **p<0.01 compared to sham rats. Analyses carried out on ~200 cells per group.

Representative images of ileum from RYGB, BMT, sham and SD rats stained for GLP-1 are shown (Fig 4A). RYGB did not significantly affect the number of GLP-1 positive cells per mm^2^ of ileum, crypt and villi (Fig 4B–4D) compared to sham. Likewise, GLP-1 remained unchanged in the SD group relative to sham. Interestingly, the BMT group exhibited a significant (p<0.01) increase in the number of GLP-1 positive cells per mm^2^ of ileum but not in the ileal crypt and villi, when compared to sham.

### PYY-cells in the ileum after RYGB or BMT

**Fig 5:**
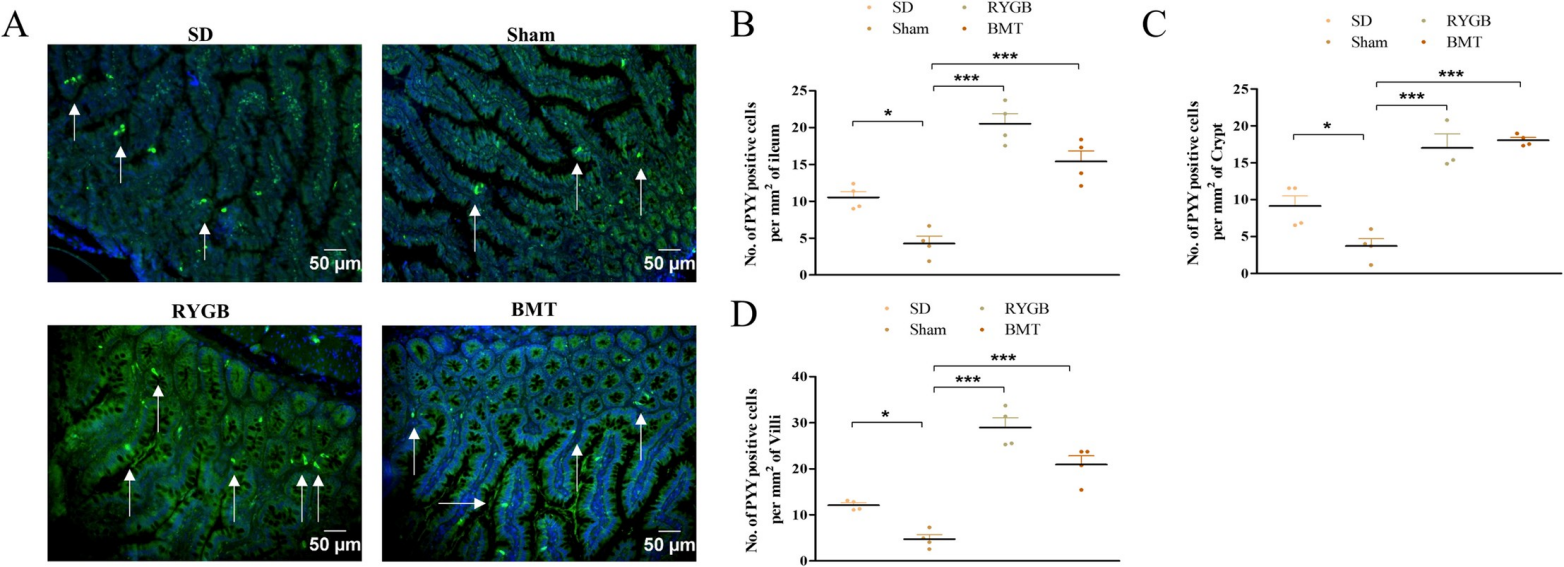
Effects of RYGB, BMT and sham surgery on PYY positive cell distribution in the ileum of ZDSD rat intestine. (A) Representative images showing PYY (green); arrows indicate PYY positive cells. Nuclei are demonstrated using DAPI staining (blue). (B) number of PYY positive cells per mm^2^ of ileum, (C) number of PYY positive cells per mm^2^ of crypt and (D) number of PYY positive cells per mm^2^ of villi analyzed in ileum sections. Normal SD rats are included for comparison. Values are mean ± SEM (n = 4) with statistical significance evaluated using one-way ANOVA. *p<0.05, ***p<0.001 compared to sham. Analyses carried out on ~200 cells per group.

Representative images of ileum from RYGB, BMT, sham and SD rats stained for PYY are shown (Fig 5A). RYGB and BMT groups exhibited a significant (p<0.001) increase in the number of PYY positive cells per mm^2^ of ileum compared to sham. SD group also had significant (p<0.05) increases in PYY positive cells compared to sham (Fig 5B). Similarly, the number of PYY positive cells per mm^2^ of crypt increased significantly (p<0.05 to p<0.001) in RYGB, BMT and SD in comparison with sham (Fig 5C). Subsequently, the number of PYY positive cells per mm^2^ of villi also significantly (p<0.05 to p<0.001) increased in groups RYGB, BMT and SD groups compared to sham (Fig 5D).

### Hormone content in the ileum after RYGB or BMT

**Fig 6:**
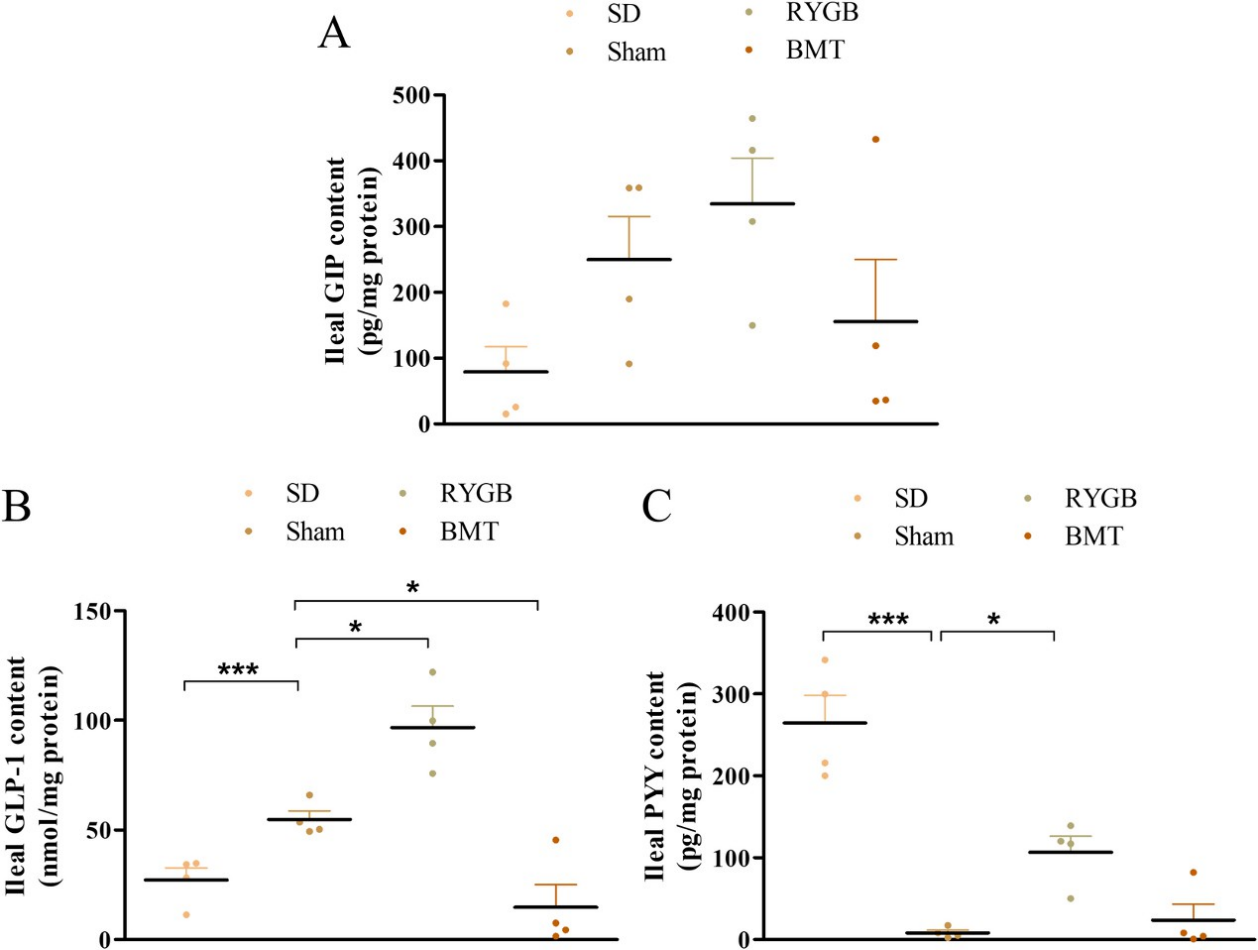
Effects of RYGB, BMT and sham surgery on ileal hormone content in ZDSD rats. (A) Ileal PYY content (pg/mg protein), (B) ileal GLP-1 content (nmol/mg protein) and (C) ileal GIP content (pg/mg protein). Normal SD rats are included for comparison. Values are mean ± SEM (n = 4) with statistical significance evaluated using one-way ANOVA. *p<0.05, ***p<0.001 compared to sham.

RYGB surgery significantly (p<0.05) increased PYY and GLP-1 content in the ileum compared with sham operated ZDSD rats (Fig 6A and 6B). However, a significant (p<0.05) decrease in GLP-1 content was observed after BMT. PYY and GIP contents did not change in the BMT group compared to sham. There was no change in GIP content in both RYGB group compared to sham (Fig 6C).

### Islet architecture and morphology after RYGB or BMT

**Fig 7:**
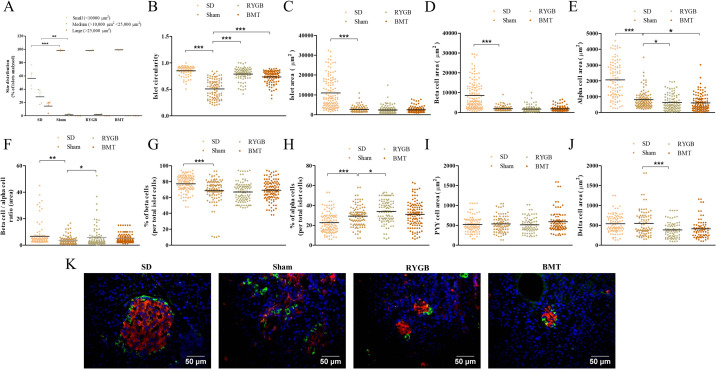
Effects of RYGB, BMT and sham surgery on islet morphology and architecture 8 weeks post-experimental intervention in ZDSD rats. (A) Size distribution (% of islets analyzed), (B) islet circularity–reports degree of roundness where 1.0 corresponds to a perfect circle, (C) islet area, (D) beta- cell area, (E) alpha-cell area, (F) beta- cell / alpha -cell ratio (area), (G) percentage of beta- cells (per total islet cells), (H) percentage of alpha-cells (per total islet cells), (I) PYY cell area and (J) delta-cell area, were determined using the ‘closed polygon’ and ‘multi-point’ tool in Olympus CellˆF analysis software. Normal SD rats are included for comparison. (K) Representative images of islets showing insulin (red), glucagon (green). Nuclei are demonstrated using DAPI staining (blue). Values are mean ± SEM (n = 4) with statistical significance evaluated using one-way ANOVA. *p<0.05, **p<0.01, ***p<0.001 compared to sham rats. Analyses carried out on 60–80 islets per group.

The sham group had significantly (p<0.001) increased number of small islets while medium islets had decreased significantly (p<0.01) compared to SD (Fig 7A). Similar to sham, RYGB and BMT groups had increased proportion of small islets and decreased proportion of medium islets (Fig 7A). There were no differences between RYGB, BMT and sham treatments and no large islets were observed in any of these groups. Islet circularity calculated to assess islet integrity, was significantly (p<0.001) greater in the RYGB and BMT groups compared to sham (Fig 7B). Islet area did not change after RYGB surgery or BMT compared to sham (Fig 7C). However, islet area in SD controls were significantly (p<0.001) higher compared to sham.

### Alpha-, beta- and delta-cells after RYGB or BMT

Beta- and alpha-cell areas both significantly (p<0.001) decreased in the sham group compared with SD controls (Fig 7D and 7E). Compared with sham operated rats, RYGB and BMT significantly (p<0.05) reduced alpha-cell area. Correspondingly, the beta- to alpha-cell ratio was increased significantly (p<0.05) in RYGB group relative to sham (Fig 7F). Like beta- cell area, RYGB did not affect the beta- cell number compared with sham (Fig 7G), but it caused a significant (p<0.05) increase in the percentage of alpha-cells (Fig 7H). PYY-cell area remained unaltered in the RYGB group (Fig 7I). RYGB caused a significant (p<0.001) decrease in the delta-cell area compared to sham (Fig 7J). Fig 7K shows representative images of pancreatic islets from RYGB, BMT, sham and SD rats.

### Co-localization of classical hormones and PYY in islets after RYGB or BMT

**Fig 8:**
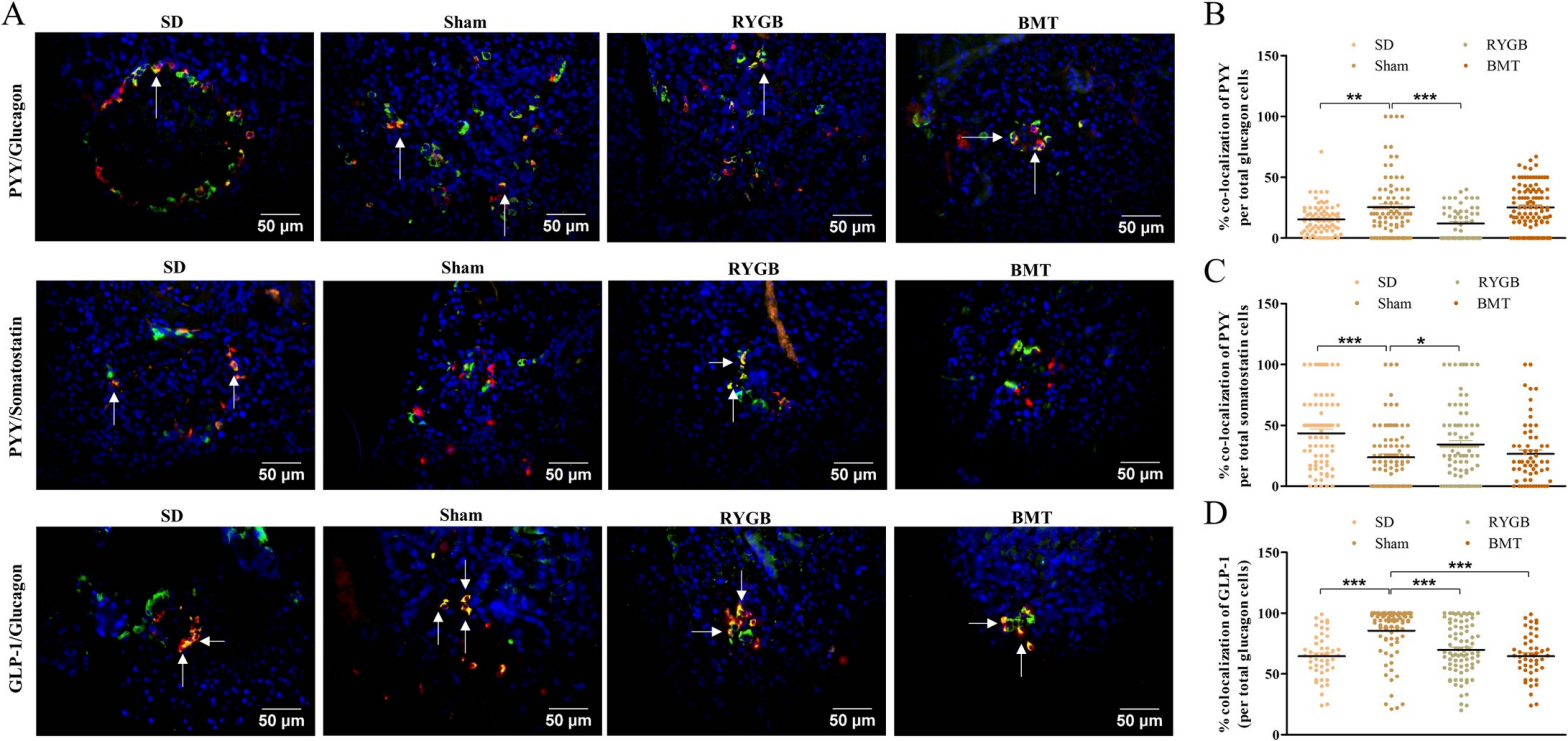
Effects of RYGB, BMT and sham surgery on gut hormone co-localization in islets 8 weeks post-experimental intervention. (A) Representative islet images showing PYY (red) with glucagon & somatostatin (green), GLP-1 (red) with glucagon (green). Nuclei are demonstrated using DAPI staining (blue). Quantification of co-localization of PYY with (B) glucagon and (C) somatostatin, and (D) GLP-1 with glucagon are also shown. Arrows indicate cells that are positive for both PYY and glucagon or somatostatin, and GLP-1 with glucagon. Normal SD rats are included for comparison. Values are mean ± SEM (n = 4) with statistical significance evaluated using one-way ANOVA. *p<0.05, **p<0.01, ***p<0.001 compared to sham rats. Analyses carried out on 60–80 islets per group.

Representative images from RYGB, BMT, sham and SD pancreatic islets stained for hormone expression are shown in Fig 8A. RYGB significantly (p<0.001) decreased the co-localization of PYY with glucagon compared to sham. A similarly significant (p<0.01) difference was observed in the SD group despite an increase in the percentage of alpha-cells (Fig 8B). However, co-localization of PYY with somatostatin significantly (p<0.05 to p<0.001) increased in the RYGB and SD groups compared to sham, although a decrease in delta-cell area after RYGB was observed (Fig 8C). Co-localization of GLP-1 with glucagon was decreased significantly (p<0.001) in RYGB, BMT and SD groups in comparison with sham (Fig 8D).

### Beta- cell proliferation and apoptosis after RYGB or BMT

**Fig 9:**
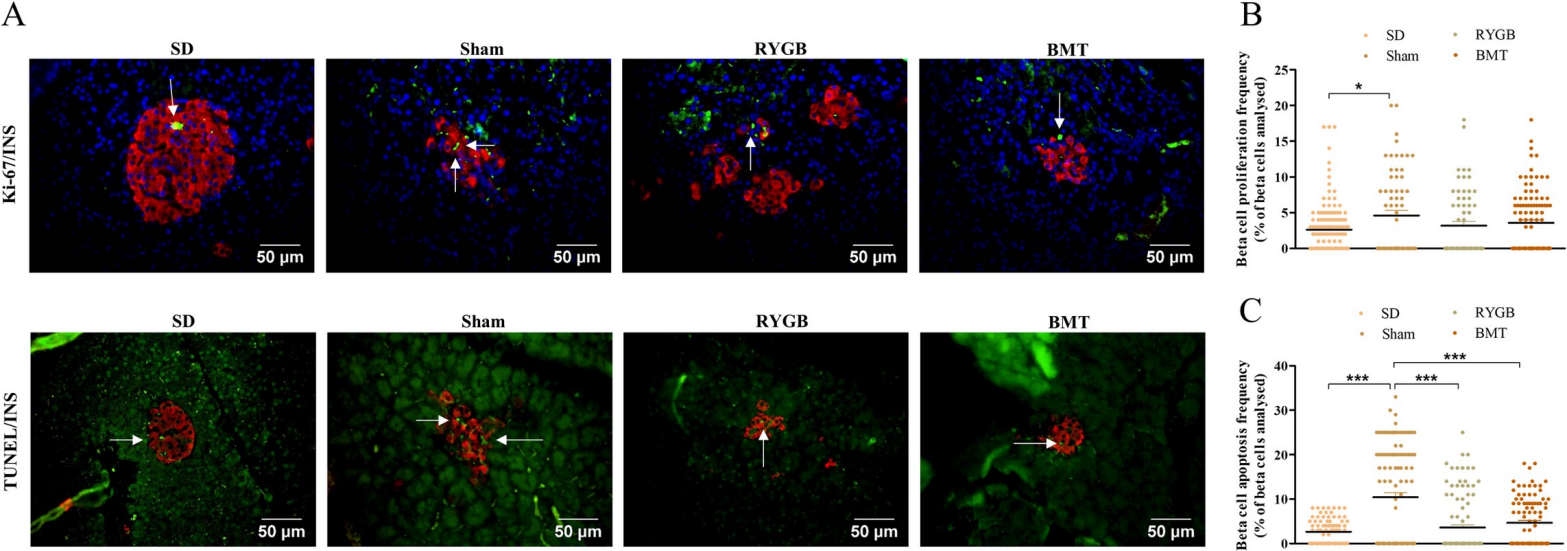
Effects of RYGB, BMT and sham surgery on beta- cell proliferation and apoptosis in ZDSD rats. (A) Representative images showing insulin (red) with Ki-67 TUNEL (green). Nuclei are demonstrated using DAPI staining (blue). (B) beta- cell proliferation frequency expressed as a percentage of beta- cells analyzed (C) beta- cell apoptosis frequency expressed as a percentage of beta- cells analyzed. Normal SD rats are included for comparison. Arrows indicate TUNEL and Ki-67 positive cells. Values are mean ± SEM (n = 4) with statistical significance evaluated using one-way ANOVA. *p<0.05, ***p<0.001 compared to sham rats. Analyses carried out on 60–80 islets per group.

Representative images of Ki-67/TUNEL and insulin-stained pancreatic islets from RYGB, BMT, sham and SD rats are shown (Fig 9A). RYGB did not influence beta- cell proliferation frequency, while it was significantly (p<0.05) lower in SD control when compared to sham (Fig 9B). Interestingly, beta- cell apoptosis frequency was significantly (p<0001) lower in RYGB, BMT and SD groups compared to sham (Fig 9C). This effect was observed despite no change in beta- cell number. A summary of these and changes in other parameters measured is presented in Table 2.

**Table 2:** Summary of results and their possible biological significance after RYGB surgery.

Parameters	Changes observed		Biological significance
	RYGB	BMT	
**Body weight**	↓	↓	Decreased risk of metabolic disease
**Plasma glucose**	↓	↓	Normoglycemia prevents risk of diabetes and related complications
**Ileum morphology/EEC distribution**			
**Crypt depth**	-	↓	Shorter crypts increase enzymatic activity and indicate efficient tissue turnover
**Villi length**	-	-	No changes observed
**GIP cell density**	-	↓	Reduction in GIP cells protect from obesity and improve lipid metabolism
**GLP-1 cell density**	-	↑	Improved satiety, gastric emptying, insulin secretion, glucose tolerance and weight loss
**PYY cell density**	↑	↑	Improved satiety, gastric emptying, islet cell function, glucose tolerance and weight loss
**GIP tissue content**	-	-	No changes observed
**GLP-1 tissue content**	↑	↓	Changes in satiety, gastric emptying, insulin secretion, glucose tolerance and weight loss
**PYY tissue content**	↑	-	Improved satiety, gastric emptying, islet cell function, glucose tolerance and weight loss
**Islet cell morphology/hormone distribution**			
**Islet circularity**	↑	↑	Increased circularity indicates functional improvements in islet paracrine interaction
**Islet area, beta cell area and number**	-	-	No changes observed
**Alpha cell area**	↓	↓	Reduction after treatments indicate a positive effect on glycaemic control
**Alpha cell number**	↑	-	Increase after surgery might be a compensatory mechanism for hyperinsulinemia and subsequent hypoglycemia
**Beta/alpha cell ratio**	↑	-	Improved insulin secretion and glucose tolerance
**PYY cell area**	-	-	No changes observed
**Delta cell area**	↓	-	Weakened inhibition of insulin and glucagon secretion
**Colocalization of classical hormones with PYY in islets**			
**PYY/glucagon**	↓	-	Reduced inhibitory effects on glucagon secretion
**PYY/SST**	↑	-	Possibly reduced inhibitory effects of SST
**GLP-1/glucagon**	↓	↓	Weakened suppression of glucagon secretion and augmentation (in hypoglycemic conditions)
**Beta cell survival**			
**Proliferation**	-	-	No change observed
**Apoptosis**	↓	↓	Improved beta cell health favouring increased beta-cell mass

Major secretory products of enteroendocrine cells (EECs)/islet cells and their associated physiological roles. Enteroendocrine K-cells secrete GIP while L-cells secrete GLP-1 and PYY. Pancreatic islets comprising major cells types that secrete glucagon/PYY/GLP-1 (alpha-cells), insulin (beta-cells), somatostatin/PYY (delta-cells). Islet and intestinal hormones act to exert a variety of physiological roles with the ultimate aim of establishing glucose homeostasis. ↑ arrows indicate significant increase, ↓ arrows indicate significant decrease and–shows no change.

## Discussion

Previous data demonstrate beneficial effects of RYGB on weight loss and remission of T2DM in both rodents and man [4, 48, 49]. As expected, we observed significant reductions in body weight and plasma glucose concentrations by 8 weeks post-surgery in ZDSD rats [34, 35]. There was a small 3–4% weight loss in the sham group compared to a 20–22% loss post-RYGB. However, sham group did not fully recover their initial body weights at 8 weeks post-surgery which may be attributed to the age-related deterioration of diabetes. Whilst weight loss is a key contributor to improved glycaemic control, recent studies suggest T2DM remission occurs partly independent of weight loss, the mechanisms behind which are largely unknown [50, 51]. This was first observed in humans and later found to be mirrored in rats where early improvements in glycaemic index, insulin resistance, beta cell function and basal metabolic rate were observed [52, 53]. A contrasting finding in rats is food intake returning to pre-surgical levels suggesting increased energy expenditure to be an important mechanism in maintaining body weight post-surgery [54, 55]. This remission has been attributed to improved beta-cell function, increased insulin sensitivity [34], enhanced post-prandial GLP-1 secretion [56] and improved glucose uptake in the gut [57]. In addition, re-routing of nutrients to distal part of the small intestine to enhance secretion of gut hormones (hindgut hypothesis) and bypassing the duodenum to trigger glucose lowering effects (foregut hypothesis), is recognised as being fundamentally important to diabetes remission [58, 59].

To investigate the role of changes in intestinal and islet morphology and function in the surgical remission of T2DM [35, 60], we utilised the well characterised obese-diabetic ZDSD rat model [61] to explore changes in gut-pancreatic axis morphology and associated hormonal adaptations in the favourable outcomes of RYGB compared with sham operated controls. Importantly, we compared the results of surgery with those of best medical treatment that comprised pre-determined optimal doses of liraglutide, metformin, rosuvastatin, ramipril and fenofibrate [46]. This regimen broadly corresponds to the type of polypharmacy commonly employed in obese T2DM subjects, with the antidiabetic agents liraglutide and metformin promoting improvements in both the secretion and action of insulin [62, 63].

Consistent with an important islet action, RYGB and BMT induced changes in the morphology of islets, improving their overall structure when compared to a distorted and fragmented shape in sham operated ZDSD rats. The morphological change was quantified using islet circularity index which suggested well-rounded islets in both experimental groups when compared to sham. Thus, in agreement with others, it is evident that RYGB, like GLP-1 therapy [64–68], restores islet morphology in rodents [20, 31, 32]. We also observed an increase in number of small islets and a corresponding decrease in large islets in all groups relative to SD rats. However, it is clear when looking at these and our other data that SD rats are an inappropriate control for obese-diabetic ZDSD rats. Thus, although derived initially from SD stock, genetic drift affecting many functional features means that the most appropriate comparator group for evaluation of the present experimental interventions is sham-operated ZDSD controls.

Compared with sham operated controls, no changes in islet or beta- cell area were observed with RYGB or BMT. This contrasts with positive effects observed in diabetic GK rats post-RYGB surgery [32] and in mouse models of obesity-diabetes treated with GLP-1 mimetics alone [69–71]. Previous *in* vitro studies have shown that GLP-1 plays a key role in beta- cell proliferation [29] but this parameter was not changed by either intervention in the present study. Consistent with our data, [31] did not find an increase in beta- cell mass after RYGB in rats. Dadheech et al. have described increases of beta- cells in hyper-insulinemic hypoglycemia after surgery [33] although such expansion of beta- cells is not consistent [72]. The lack of effect of surgery on beta- cell proliferation is consistent with results in GK rats [32, 72], but we did observe a substantial reduction in beta- cell apoptosis with both the RYGB and BMT groups.

Interestingly, a significant reduction in islet alpha-cell mass was observed following RYGB and BMT, resulting in favourable increases in beta-alpha-cell area ratio in the RYGB group. However, new increased clusters of alpha-cells were observed in the RYGB group suggesting islet adaptation or alpha-cell neogenesis to help counter the development of hyperinsulinemia and post-prandial hypoglycemia which can sometimes be a side effect of surgery. Contrary to our results, no alterations in alpha-cell mass or glucagon staining islet cells were observed by others after RYGB surgery [20, 32, 35] whilst recent study by Pérez-Arana et. al observed increased alpha cell mass after RYGB [73]. The reasons for this are unclear but may be related to the different animal models used, their age and effects on islet cell transitioning events. In this respect, it is interesting that both liraglutide and metformin have been shown to exert positive effects on islet cell trans differentiation [74].

PYY which is present in both the intestine and various sub-types of islet cells, has been implicated in ameliorating diabetes in rodents and man after RYGB surgery [75–78]. Studies have also highlighted its role in negatively modulating insulin secretion and preserving beta- cell function [29, 79–81]. Our analysis in islets showed decreased co-expression of PYY with glucagon after RYGB surgery with no change in overall islet PYY-cell area compared with sham operated ZDSD rats. This coincided with a decrease of alpha-cells and there was increased co-expression of PYY with somatostatin despite a RYGB-induced decrease in delta-cell area. No such changes were observed following BMT suggesting that part of the distinction between the two interventions in remission of diabetes in man may be due to differential effects on islet cell populations and their ensuing autocrine or paracrine interactions. Thus, changes in the secretory products of one islet cell may exert important beneficial effects on the functional activity of that cell or those of its immediate neighbours.

Further studies are required to assess functional interactions between islet cell populations following RYGB and possible involvement of loss of somatostatin-induced inhibitory tone from delta-cells in insulin-induced hypoglycemia [82–85]. Interestingly, studies from glucagon receptor knockout mice show suppression of delta-cell mass [86]. Guida and colleagues (2018) also found co-localization of PYY within delta-and PP-cells whilst no PYY was expressed in rat alpha-cells [87]. To better understand the effect of PYY, we studied its distribution post-RYGB in L-cells, a major site of synthesis in the distal intestine. There was a significant increase in the number of PYY positive cells in the ileum after surgery and BMT. The immunostaining in the crypts and villi were both increased, possibly reflecting strong positive effects of both interventions on satiety and gastric emptying. We also observed a significant increase in PYY tissue content in the ileum after RYGB compared to sham. These observations are broadly consistent with previous studies [76] and the demonstration of elevated post-prandial PYY with peak concentrations which precede those of GLP-1 [20, 76, 88–91]. However, observations with PYY and NPY2 receptor null mice have been less convincing regarding involvement of PYY in the positive effects of RYGB surgery [22, 92]. Taken together, the distribution of PYY and its adaptation within the gut and islets following RYGB merit further investigation.

We observed decreased co-expression of GLP-1 with glucagon in islets of the RYGB and BMT groups compared to sham. Previous studies have demonstrated GLP-1 in alpha-cells in rodent and human islets, with the PC1/3 derived processing product of proglucagon being increased under conditions of diabetes and islet stress [41, 93–95]. Hence, reduced GLP-1 secreting alpha-cells observed in the present study might be an indication of improved beta- cell health. A recent report of an increase in GLP-1 receptor positive cells in islets of GK rats after RYGB surgery may thus reflect a more ongoing active role in islet repair [34]. It is of note here that excess GLP-1 has been proposed as a driving factor for hyperinsulinemia-induced hypoglycemia sometimes encountered after RYGB [96]. Intestinal derived GLP-1 may contribute to such an effect but in the present study RYGB rats showed a slight but non-significant enhancement of GLP-1 positive cells compared to a prominent increase following BMT. However, ileal GLP-1 tissue content was decreased with BMT, whereas it was significantly increased following RYGB surgery. This accords with recent studies reporting increased numbers of GLP-1 positive cells in the ileum, duodenum, jejunum as well as gastric mucosa of rats after RYGB surgery [76, 97, 98]. Like PYY, studies using GLP-1 receptor knockout or exendin (9–39) have presented varying results on whether GLP-1 is a key mediator of the metabolic benefits in RYGB, calling for further exploration [42, 99–101].

Following GLP-1, we found no change in ileal GIP content after RYGB surgery and correspondingly unchanged numbers of GIP positive cells in the ileum compared to sham. In contrast, GIP content was significantly decreased in the ileum of the BMT group. Consistent with our study, GIP positive cells have been reported to be unchanged in the rat ileum following RYGB [76]. Interestingly, Zhou et al. [102] found that GIP was selectively decreased in the Roux limb but not in the other surgical limb possibly due to differences in exposure to undigested nutrients, reflecting the unavailability of bile acids and pancreatic enzymes which play key roles in the digestion and absorption of carbohydrate, protein and fat. Also post-RYGB, there can be a reduced consumption of dietary lipids that, like bile acids, normally serve as potent stimulators of GIP secretion. A possible explanation for unchanged ileal GIP content observed in our study might be lesser exposure to lipids from the Roux limb, thereby masking a decrease in GIP. To support this view, we found a decrease of GIP cells in the BMT ileum which is not subjected to altered nutrients supply. Changes in GIP secretion following RYGB surgery are not altogether clear with decrease, increase and no change being reported [18, 43, 97, 103]. Thus, the significance of GIP cell function after RYGB remains to be determined as does the decreased ileal GIP cell population following BMT. It is noteworthy that transcriptomic and peptidomic profiles of human and mouse EECs were unaltered post-vertical sleeve gastrectomy (VSG) in a study by Larraufie et al. [15]. This might reflect extraction from *in- vivo* hyperglycemia and insulin resistance which would otherwise pose a stressful environment for compensatory post-surgery mechanisms by EECs. Importantly, we saw no significant differences in ileal villus length or crypt depth following RYGB in agreement with [104], whereas the latter parameter was decreased by BMT. While others have reported increased villi length and crypt depths after RYGB surgery [104–108], this disparity could be due to the different time points of assessments after surgery or the variance in animal models and study design. Finally, we did not observe any hypertrophy of villi or crypts as in previous studies where elongated villi, deeper crypts and mucosal hypertrophy was reported [107, 108]. It remains to be elucidated whether changes in the secretory capacity of EECs are involved.

Taken together, this study uncovers morphological and functional adaptations of islet and intestinal cell hormones following RYGB surgery. Comparison with the effects of BMT reveal striking similarities but also some important differences, particularly regarding islet cell composition and paracrine interactions, that might underlie the unique ability of RYGB surgery to cause diabetes remission. Our data, summarized in Table 2, call for further detailed studies of the mechanisms through which alterations of incretin hormones affect glucose control, insulin/glucagon secretion and sensitivity in RYGB and BMT. A better understanding of the mechanisms involved in this phenomenon will not only represent a step change in our understanding of glucose regulation but hopefully also pave the way for cost-effective and less-invasive pharmaceutical treatment of obesity and T2DM.
